# Construction and Bioengineering of Human Bioprosthetic Ovaries from Cryopreserved Ovarian Tissue

**DOI:** 10.3390/ijms26125545

**Published:** 2025-06-10

**Authors:** Mengyang Cao, Plamen Todorov, Gohar Rahimi, Mahmoud Salama, Teresa K. Woodruff, Evgenia Isachenko, Christine Skala, Volodimir Isachenko

**Affiliations:** 1 Department of Obstetrics and Gynecology, Medical Faculty, Cologne University, 50931 Cologne, Germany; weimusjtu@gmail.com (M.C.); evgenia.isachenko@uk-koeln.de (E.I.); christine.skala@uk-koeln.de (C.S.); 2 Institute of Biology and Immunology of Reproduction of Bulgarian Academy of Sciences (BAS), 1113 Sofia, Bulgaria; plamen.ivf@gmail.com; 3 AMEDES Facharzt-fZentrum für Kinderwunsch, Pränatale Medizin, Endokrinologie und Osteologie GmbH, 90518 Hamburg, Germany; gohar.rahimi@amedes-group.com; 4 Department of Obstetrics, Gynecology and Reproductive Biology, College of Human Medicine, Michigan State University, East Lansing, MI 48824, USA; salamam2@msu.edu (M.S.); tkw@msu.edu (T.K.W.)

**Keywords:** ovarian tissue cryopreservation, bioprosthetic ovaries, follicle loss, revascularization, scaffolds

## Abstract

Ovarian tissue cryopreservation is increasingly recognized as an effective fertility preservation option for cancer patients undergoing gonadotoxic therapies. After cancer treatment, transplantation of frozen–thawed ovarian tissue can restore both fertility and endocrine function. However, the threat of reintroducing malignant cells limits its application in patients with a high risk of ovarian metastasis. To eliminate potential cancer cells in grafts, a promising strategy involves isolating follicles from cryopreserved ovarian tissue and encapsulating them within biocompatible scaffolds to construct transplantable bioprosthetic ovaries. Here, we review the construction of bioprosthetic ovaries designed to mimic natural ovarian architecture, and further discuss the challenges in bioprosthetic ovary bioengineering along with potential strategies to address these issues.

## 1. Introduction

As cure rates for cancers have improved, there has been an increasing awareness of the fertility consequences for young patients after cancer therapies. Female cancer survivors often face a high risk of primary ovarian insufficiency after gonadotoxic treatment like chemotherapy and radiotherapy, which makes their fertility preservation emerge as a significant issue [1]. Currently, the management of fertility preservation focuses on three primary clinical approaches: embryo cryopreservation, oocyte cryopreservation, and ovarian tissue cryopreservation [2]. Among these, ovarian tissue cryopreservation is the only feasible fertility preservation method for prepubertal girls and women who cannot postpone their gonadotoxic treatment [2,3]. Beyond restoring fertility, this technique uniquely offers the potential to reestablish ovarian endocrine function, which is considered to have long-term health benefits for cancer survivors [2,3]. Since the first live birth from cryopreserved ovarian tissue in a Hodgkin’s lymphoma patient in Belgium, this promising technology has significantly advanced over the past decades [4]. In 2011, despite ovarian tissue being transported on ice overnight before freezing, a successful live birth was achieved in Germany after transplantation [5,6]. By 2020, it is estimated that over 200 live birth cases following transplantation of cryopreserved ovarian tissue had been reported [7]. An analysis of data from 60 patients across three major European fertility centers in 2020 demonstrated a 50% pregnancy rate and a 41% live birth rate, confirming the safety and efficacy of ovarian tissue cryopreservation as a fertility preservation method [8]. In light of this compelling evidence, the American Society for Reproductive Medicine recommended in 2019 that ovarian tissue cryopreservation should no longer be considered experimental but rather an established fertility preservation technique [9].

However, cryopreserved ovarian tissue from cancer patients with ovarian involvement may contain malignant cells. Transplanting such tissue exposes cancer survivors to a significant risk of reintroducing malignant cells and disease recurrence, thereby limiting the clinical application of ovarian tissue cryopreservation [10,11]. Among adult patients, hematologic cancers (Hodgkin’s lymphoma, non-Hodgkin’s lymphoma, and leukemia) and breast cancer are the leading indications for ovarian tissue cryopreservation [2]. For pediatric patients, one study characterized indications of 114 girls who underwent ovarian tissue cryopreservation and showed that hematologic neoplasms and soft tissue sarcomas occupied half of the cases [12]. Given the heightened risk of chemotherapy-induced ovarian reserve damage in young cancer survivors, it is crucial to assess the likelihood of ovarian metastasis across different tumor types before proceeding with clinical interventions. In a related study, 12 ovarian tissue slices from patients with acute leukemia were transplanted to immunodeficient mice, and finally, one-third of these mice developed intraperitoneal leukemic masses after six months [10]. According to a report by Dolmans and Masciangelo, leukemia, neuroblastoma, and Burkitt lymphoma are classified as high risk (more than 10%) for ovarian metastasis, while advanced breast cancer, non-Hodgkin’s lymphoma, and Ewing sarcoma are categorized as moderate risk (2–10%) [13]. For patients with these high-risk conditions, the potential transfer of malignant cells during transplantation of cryopreserved ovarian tissue remains a critical concern.

Many treatment strategies have been proposed to address this challenge. These strategies are primarily categorized into two approaches: isolating follicles from frozen–thawed ovarian tissue for in vitro maturation or encapsulating them in transplantable bioprosthetic ovaries for in vivo growth [2,14,15]. However, folliculogenesis is a complex and dynamic process whose underlying mechanisms remain incompletely understood; thus, it is difficult to establish a suitable management of in vitro culture. Beyond this, it takes about one year during human folliculogenesis from primordial follicle activation to the culmination of ovulation, and interference with oocytes’ epigenetic status after a long time in vitro culture has to be considered [14]. Notably, improper in vitro culture conditions can disrupt genomic imprinting, potentially leading to genetic disorders such as Prader–Willi and Angelman syndromes [16]. For these reasons, encapsulating isolated follicles into scaffolds to create bioprosthetic ovaries is considered a safer alternative to reduce the potential risk of cancer cells inclusion. In this paper, we review the procedures for constructing bioprosthetic ovaries from cryopreserved ovarian tissue and further discuss bioengineering strategies of bioprosthetic ovaries for potential applications.

## 2. Construction of Bioprosthetic Ovaries from Cryopreserved Ovarian Tissue

**Figure 1:**
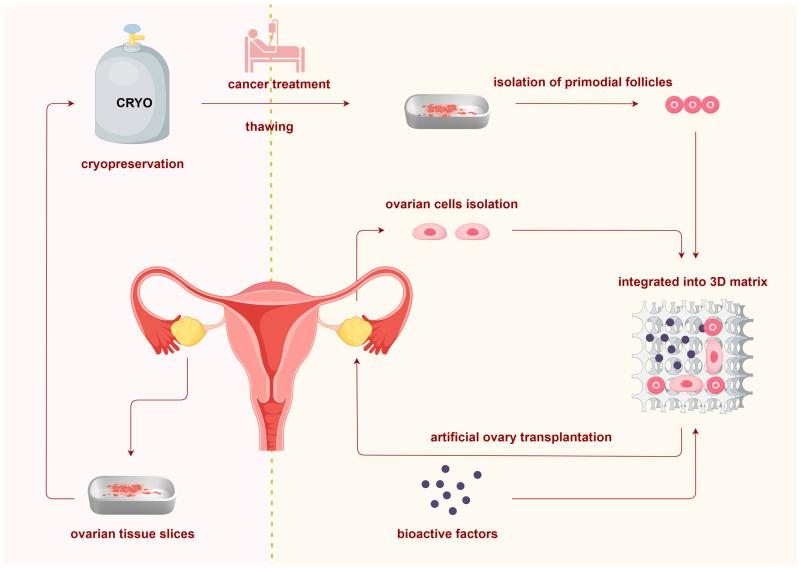
Overview of bioprosthetic ovary construction from cryopreserved ovarian tissue. In cases where cancer patients require immediate treatment or are prepubertal, ovarian tissue is surgically removed and cryopreserved via slow freezing. After cancer treatment, ovarian metastasis is evaluated to assess the safety of cryopreserved tissue for transplantation. If there is a risk of transmitting malignant cells, follicles are isolated from thawed tissue. These isolated follicles, along with ovarian stromal cells from fresh biopsies and bioactive factor particles, are encapsulated within scaffolds to create bioprosthetic ovaries, which can then be transplanted as an alternative to the original cryopreserved tissue.

Bioprosthetic ovaries are designed to prevent cancer recurrence during the ovarian tissue cryopreservation and transplantation process. In practice, if patients are prepubertal or require immediate gonadotoxic therapy, ovarian tissue cryopreservation is the only fertility preservation approach [2,3]. Typically, ovarian tissue is surgically removed and cryopreserved through slow freezing before the initiation of gonadotoxic treatment [17]. It is also recommended that original tumor samples be cryopreserved alongside ovarian tissue for future cancer analysis [13,18]. After cancer treatment is completed, the first step in determining appropriate therapeutic options is a risk analysis of ovarian metastasis [13,19]. Many methods have been used to estimate the risk of ovarian metastasis, including identifying disease-specific markers in specimens by immunohistochemistry and grafting thawed tissue slices into severe combined immunodeficient mice, although no standardized practice exists to date [10,20]. If the risk of malignant cell transmission cannot be ignored, follicles can be isolated from thawed tissue and encapsulated in a matrix. To mimic the physical and biological characteristics of natural organs, such a matrix will also integrate additional ovarian cell populations and bioactive factors essential for folliculogenesis [19]. Finally, bioprosthetic ovaries are grafted to patients and are desirable to maintain follicle survival until replaced by a developed natural architecture. In conclusion, bioprosthetic ovaries generally include four interrelated components: isolated follicles, ovarian stromal cells, matrix, and bioactive factors. Figure 1 presents an overview of the bioprosthetic ovary construction process, from ovarian tissue cryopreservation to transplantation.

### 2.1. Isolation of Follicles from Cryopreserved Ovarian Tissue

Achieving sufficiently large numbers of follicles is the first step for bioprosthetic ovary construction, which requires dissociating as many follicles as possible from the surrounding ovarian cells and matrix. Many methods have been developed to obtain follicles from cryopreserved human ovarian cortex, with a combination of mechanical and enzymatic digestion being the most effective approach to date [21]. However, there is still no consensus on which protocol will yield the best results. Vanacker et al. chose Liberase DH as the digestion enzyme and showed that it performed better than traditional Collagenase in obtaining follicles and maintaining their viability [22]. Given the potential damage caused by prolonged enzyme exposure to fully isolated follicles, Chiti et al. optimized the previous protocol by dividing the digestion process into short, repeated cycles [23]. In their optimized isolation protocol, the ovarian tissue incubation process is terminated every 30 min, followed by filtration of the digestion suspension. After obtaining the first isolated follicles, the remaining tissue is incubated in digestion media to start a new cycle until the ovarian tissue is completely digested. Their study demonstrated that this personalized procedure was able to improve the quality and quantity of isolated primordial follicles. An attractive alternative agent is Tumor Dissociation Enzyme, which has proven to be effective without inducing additional apoptosis and necrosis in follicles [24,25,26]. Beyond these, other digestive enzymes like collagenase NB6 and Liberase TM have also been tried to maximize follicle acquisition efficiency [27,28]. In conclusion, individualized isolation protocols are worthy of further investigation to achieve a better outcome.

### 2.2. Isolation of Ovarian Stromal Cells

Ovarian stromal cells provide the architectural framework for the ovary and support early follicle development through a complex network of interacting paracrine, hormonal, and neuronal signaling pathways. After activation, ovarian stromal cells adjacent to follicles are modulated and recruited to differentiate into functional theca cells [29]. Thus, integrating ovarian cells into bioprosthetic ovaries and co-transplanting them with follicles is considered an optimal approach to better simulate the three-dimensional ovarian microenvironment. Moreover, given the critical role of endothelial cells in angiogenesis, co-transplanting ovarian endothelial cells along with stromal cells has been shown to promote vascularization and the formation of structured, ovary-like tissue [30]. Similarly, another study demonstrated that high concentrations of endothelial cells isolated from the medulla can enhance vascularization in transplanted tissue [31]. Considering the potential risk of reintroducing malignant cells, ovarian cells should preferably be isolated from fresh ovarian biopsies taken after cancer treatment is complete, rather than from cryopreserved fragments. The impact of previous chemotherapy or radiotherapy is a possible concern, but evidence suggests that gonadotoxic cancer treatment does not appear to have significant negative effects upon isolated ovarian cells [31]. Therefore, obtaining stromal cells from patients after successful cancer treatment could be a promising safe and effective approach.

### 2.3. Matrix

It is important to note that the matrix is not merely a passive container; it actively interacts with bioactive components and shapes the follicle microenvironment [32,33]. An ideal matrix should replicate both the anatomical and functional features of natural organs to support follicle survival in the early stages of post-transplantation [34]. Therefore, specific principles need to be considered in matrix design. Firstly, the matrix is expected to exhibit good biocompatibility with surrounding tissues, which implies that it should be non-toxic and not elicit a sustained local or systemic immune response [34,35]. In addition, its degradation products should be non-cytotoxic and easily eliminated through metabolic pathways. Secondly, the physical properties of the matrix must provide temporary structural and mechanical support to sustain essential biological activities [36]. Appropriate material porosity and strength are critical to facilitate cell behaviors such as adhesion, proliferation, migration, and differentiation [32,36]. Moreover, the matrix architecture should enable the efficient delivery and controlled release of biological components such as growth factors, hormones, and stem cells to specific targeted sites [36,37]. Finally, proper biodegradability also plays an important role in scaffold selection. The size of reserved follicles ranges from 30–50 μm in the primordial stage to 100–200 μm during the preantral phase, with Graafian follicles expanding up to approximately 18 mm in diameter—an increase of about 600-fold [38,39]. Therefore, the scaffold must have a suitable degradation rate that accommodates changes in cell size, maintaining follicle morphology until the scaffold is gradually replaced by functional tissue [40].

Plasma clots were probably the first natural materials tested for encapsulating isolated murine follicles. In 1990, Gosden reported the potential of using plasma clot-based grafts to produce offspring by integrating digested mouse ovarian tissue into plasma clot scaffolds and transplanting them to sterile mice [41]. Shortly after, Carroll and Gosden further demonstrated that cryopreserved murine ovarian tissue yielded similar results to fresh ovarian samples, although the lifespan of the grafts was significantly limited [42]. Building on these studies, Dolmans et al. extended the work from mice to human tissue with promising results. Their studies proved that isolated human follicles encapsulated in autologous plasma clots can grow up to the secondary stage after one-week xenograft and survive for up to five months [43,44]. However, this approach requires the injection of fresh human autologous plasma with isolated follicles under direct vision to form a clot, which is inconvenient to further handle and highly prone to degradation [43]. Moreover, the complex composition of plasma poses challenges for future research and practical applications, as it may introduce unknown factors that could indirectly contribute to follicle loss. With advances in biomaterials, several scaffold materials have been investigated to replace plasma clots. Based on their different manufacturing processes and material sources, scaffold-based substrates are grouped into the following three categories: natural polymers, decellularized extracellular matrix (dECM), and synthetic polymers. To fully leverage the benefits of various biomaterials, composite scaffolds combining two or more materials have also been developed.

#### 2.3.1. Natural Polymers

Fibrin is a widely used natural polymer in the field of tissue engineering. During coagulation, fibrinogen is converted into fibrin under thrombin catalysis, after which factor XIIIa promotes fibrin cross-linking until subsequent stabilization to form a fibrin clot [45,46]. The physical properties of the fibrin network like elasticity and porosity can be adjusted by varying the concentrations of fibrinogen and thrombin [45,47]. Fibrin is also capable of conveying biological macromolecules to accomplish a wide range of specialized functions, such as fibrinolysis inhibitors for decreasing the degradation [48,49]. Additionally, fibrin is highly compatible with other natural and synthetic polymers, allowing for the synthesis of composites depending upon specific requirements [45,46]. Overall, fibrin can be easily and functionally modified to serve various purposes, which provides an ideal platform for bioengineering scaffolds in encapsulating follicles [50].

Studies by Luyckx et al. reported the survival and growth of isolated murine ovarian follicles in fibrin-based scaffolds after short-term (1 week) autotransplantation for the first time [35,47]. A subsequent study demonstrated that fibrin-based grafts facilitated host cellular infiltration and ultimately restored ovarian function in infertile mouse models over an extended period (3 weeks) [51]. Additionally, the influence of the fibrin matrix on follicle survival varied across different follicular stages, with primordial primary follicles exhibiting lower recovery rates than secondary follicles in mice [52]. This disparity may be attributed to the absence of factors required for early follicular development in the scaffolds. Consequently, fibrin has been modified with bioactive factor loading to improve follicle survival. After extracting primordial follicles from young female mice and transplanting fibrin-encapsulated follicles into infertile mice, Kniazeva et al. demonstrated that while all infertile mice resumed cycling, live births were achieved only when the transplanted follicles were encapsulated in fibrin hydrogels loaded with vascular endothelial growth factor (VEGF) [53]. Moreover, fibrin hydrogels supplemented with platelet lysate were shown to enhance angiogenesis in grafts encapsulating cryopreserved preantral follicles [54].

Based on these results from animal studies, several groups have further investigated the potential application of fibrin for encapsulating human follicles. Since human ovarian tissue is more rigid than murine tissue, higher concentrations of fibrinogen and thrombin are required to provide adequate structural support for grafting isolated human preantral follicles [55]. To determine the optimal fibrin formulation that best mimics natural human ovarian tissue, Chiti et al. conducted tests on material architecture, porosity, and rigidity using various fibrinogen/thrombin (F/T) concentration combinations. Their findings indicated that F50/T50 most closely resembled the ultrastructure and rigidity of the human ovarian cortex [56]. However, the high degradability and low rigidity remain significant drawbacks of fibrin-based scaffolds, which often lead to insufficient structural support before adequate cell proliferation and tissue regeneration [45,46,57].

Alginate is a linear polysaccharide derived from brown algae known for its good biocompatibility, easy accessibility, and capacity for encapsulating cells, which makes it a desirable option for ovarian tissue engineering [58]. To date, alginate has been used primarily as a matrix for studying follicle development in vitro rather than for encapsulating isolated follicles [59,60,61]. In an early attempt to transplant an alginate–Matrigel matrix containing isolated ovarian cells, Vanacker et al. were the first to entrap murine follicles into an alginate-based scaffold with ovarian cells. One week after grafting, roughly one-third of the follicles were recovered, with some reaching more advanced stages of development, while the alginate beads were invaded by proliferating murine cells [62,63]. Subsequently, a study by Rios et al. investigated the fertilization potential of surviving follicles in alginate grafts. Surviving follicles were fertilized via intracytoplasmic sperm injection and demonstrated the ability to develop into two-cell and four-cell stages following standard in vitro fertilization procedures [64]. However, the biodegradation of alginate hydrogels is typically slow and difficult to control, which may negatively impact further follicle development [58,65]. Additionally, the lack of cell-binding sites presents another significant limitation, and modifying alginate with other polymers, such as extracellular matrix proteins, fibrin, and gelatin, may be a potential solution [66,67,68].

In addition to fibrin and alginate, various other natural polymers have been investigated as potential scaffolds. Gelatin was used as a bioink by our coauthor, Teresa K. Woodruff’s team, to create bioprosthetic ovaries with three-dimension (3D) printed microporous scaffolds. After implanting follicle-seeded scaffolds in surgically sterilized mice, the animals successfully restored normal ovarian function and produced offspring through natural mating [69]. Another study by Wu et al. investigated various natural polymers as bioinks for 3D printing bioprosthetic ovaries and concluded that gelatin-methacryloyl scaffolds exhibited superior mechanical properties while providing a suitable microenvironment for ovarian follicles [70]. These findings form a foundation for the broader application of 3D printing in ovarian tissue engineering. Zand et al. demonstrated that a composite scaffold comprising Human Wharton’s Jelly and alginate could support both in vitro and in vivo development of encapsulated murine preantral follicles up to the antral stage. Moreover, Human Wharton’s Jelly enhanced the expression of factors contributing to angiogenesis in transplanted encapsulated follicles [71]. In addition, agarose, chitosan, and hyaluronic acid are also considered promising hydrogels for follicle culture; however, limited data currently prevent drawing definitive conclusions about their effects on follicle outcomes post-transplantation [36].

#### 2.3.2. Decellularized Extracellular Matrix

More recently, dECM-based scaffolds have become a central focus in the fields of tissue engineering and regenerative medicine. By preserving the structure and characteristics of natural tissue, dECM provides a specialized microenvironment conducive to the recellularization process [72]. In addition, dECM retains growth factors and cytokines essential for native tissue regeneration, which makes it an ideal candidate for creating engineered organs [72,73]. To date, dECM-based scaffolds have been successfully employed to functionally repair and regenerate complex tissues and organs, including the kidney, liver, and heart [74,75,76]. Commercially available dECM reagents have also been used in ovarian tissue transplantation [77]. Building on previous research into decellularized tissues, improvements in the decellularization protocol for ovarian tissues have yielded promising results. Laronda et al. were the first to decellularize bovine ovaries and recellularize them with murine ovarian cells to create grafts, which finally initiated puberty in ovariectomized mice after transplantation [78]. Similarly, a study by Pors et al. demonstrated that isolated human follicles could survive in decellularized human ovarian tissue scaffolds after long-term transplantation [79].

Despite these substantial advances, practical applications remain limited by several significant drawbacks. Given that dECM scaffolds can only be made from human tissue, obtaining sufficient quantities for patients remains a major obstacle. Ovarian tissues obtained from donors may represent a viable alternative source. However, the immunological response to allogeneic ovary-derived dECM scaffolds remains unexplored in current research. One study involving human vascular tissues demonstrated the absence of an immune cell response to vascular-derived dECM, suggesting a potentially reduced immune reaction in vivo and supporting the feasibility of using allogeneic tissues for replacement [80]. Conversely, another study reported that immune rejection can also occur following allogeneic transplantation of decellularized uterine scaffolds in rats, with the severity of the response influenced by the specific decellularization protocol employed [81]. Therefore, the potential risk of follicle loss resulting from immune rejection remains a critical concern that should not be underestimated. Moreover, a large number of ovarian cells and isolated follicles are also required for the effective recellularization of dECM. Finally, the low stiffness and rapid degradation of dECM remain challenging for the current decellularization methods [65,82].

#### 2.3.3. Synthetic Polymers

Synthetic polymers are high-molecular-weight macromolecules composed of covalently bound repeating units. Compared to natural polymers, synthetic polymers are easily manufactured on a large scale with controlled degradation and mechanical properties [65]. Kim et al. were the first to test a synthetic hydrogel, poly(ethylene glycol) vinyl sulfone (PEG-VS), as a supportive matrix by orthotopically implanting primordial follicles encapsulated in PEG-VS hydrogels into ovariectomized mice. The results showed that PEG-VS-based grafts were sufficient to support folliculogenesis 30 days post-transplantation and partially restored ovarian functionality for at least 60 days in vivo [83]. This is also the only synthetic material reported to support follicle development to date. Synthetic polymers have also been combined with other materials to yield enhanced performance. For instance, biocompatible PEG can be attached to the targeted structure of fibrin to improve mechanical strength and reduce proteolysis [84,85]. A study by Dadashzadeh et al. indicated that PEGylated fibrin hydrogels with a proper ratio can support the viability and proliferation of human ovarian stromal cells in vitro, which may be considered as a potential candidate to serve as a matrix for the encapsulation of isolated human follicles [86]. However, the lack of intrinsic biocompatibility and concerns regarding the potential adverse effects of degradation products limit the applications of synthetic polymers [87,88]. The main results of the studies mentioned above that address outcomes of isolated follicles after transplantation are summarized in Table 1.

The ultimate goal of human bioprosthetic ovaries is to restore both endocrine and reproductive functions after grafting, like natural tissues. As shown in Table 1, several prototypes have demonstrated the ability to support follicle survival and development in the short term (within two weeks) following transplantation in both murine and human models. Some previously mentioned studies have evaluated long-term outcomes—such as ovulation, synthesis of female hormones, and the generation of healthy embryos and offspring [41,42,51,53,69,78,83]. While findings obtained from murine models using these matrices may offer valuable insights, it is critical to acknowledge the substantial differences in ovarian microarchitecture and physiology between mice and humans. Consequently, the design criteria and functional requirements for human bioprosthetic ovaries are likely to diverge significantly from those optimized for murine systems. In current experimental studies, the longest reported duration of transplantation for human bioprosthetic ovaries is five months by Dolmans et al.; however, no indicators of general ovarian function—such as hormone production, ovulation, or the generation of offspring—have been observed during this period [44]. To date, long-term experimental data on the transplantation of human bioprosthetic ovaries remain significantly more limited than those in murine models, which may be attributable to factors such as tissue availability or ethical considerations. To further understand the underlying interactions between selected matrices and cell populations, evaluating the long-term functional maintenance of grafted bioprosthetic ovaries represents an important direction for future research.

### 2.4. Additional Bioactive Components

Compared to natural ovarian tissue, the matrix of bioprosthetic ovaries is convenient to modify according to the clinical needs, potentially providing opportunities for follicles and ovarian cells to benefit from added bioactive components. In practice, grafts often experience hypoxia and ischemia before the onset of angiogenesis and perfusion [89]. Therefore, the supplementation of bioactive components is considered valuable to support follicle survival and reduce injury from dysmetabolism during the early post-transplantation period [89,90]. Several bioactive factors, hormones, and stem cells have demonstrated the ability to improve follicle reserve, and the matrix of bioprosthetic ovaries is proposed to function as a drug-delivery vehicle. However, human folliculogenesis is a complex developmental process involving the coordinated interplay of numerous molecules, many of which remain poorly understood. As a result, identifying the key factors involved in the initial growth of primordial follicles and their metabolism under hypoxic conditions is challenging. Another critical issue is integrating bioactive factors into bioprosthetic ovary scaffolds in a manner that preserves their activity and prevents premature degradation. Ideally, these factors should be released in a controlled manner, responding dynamically to the demands of different phases of the cell cycle. Addressing these challenges could provide valuable insights into optimizing bioprosthetic ovary design, which we discuss in detail in the following sections.

## 3. Challenges in Bioprosthetic Ovary Construction: Safety and Efficiency

Although earlier studies have demonstrated the feasibility of bioprosthetic ovaries, their potential application still faces two main challenges. The foremost concern is the safety of this novel method, including the possible contamination of cancer cells during bioprosthetic ovary construction and unpredictable effects on the epigenetic status of oocytes. Another critical problem is the substantial loss of follicles following transplantation, which significantly reduces the capacity to restore ovarian reserve. Therefore, improving the efficiency of follicle survival during the early post-graft stage is another point to consider.

### 3.1. Safety Concerns in Bioprosthetic Ovary Construction

As previously stated, isolation of follicles is the key step to prevent the reintroduction of malignant cells present in cryopreserved tissue. Although follicles are encased in a layer of fibrous membrane that could hinder cancer cell invasion, the risk of retrieving cancer cells still exists during the follicle collection process. Previous studies have shown the presence of residual cancer cells in medium droplets containing isolated follicles from leukemia patients [91,92]. In order to prevent possible cancer cell contamination, a three-time wash process following the follicle collection is recommended to completely eliminate any residual cells [91,92]. In a subsequent study, a small number of leukemic cells were embedded in bioprosthetic ovaries with normal follicles and then grafted into immunodeficient mouse xenografting models. However, none of the mice showed any signs or symptoms of leukemia recurrence after 20 weeks [93]. This result implies that although small amounts of malignant cells may also enter the graft, the limited quantity is not sufficient to cause cancer. Overall, existing studies have demonstrated the safety of the bioprosthetic ovary method, but validation on a larger scale is still required before clinical application. Despite the risk of tumor recurrence, the epigenetic status of oocytes in fertility preservation is receiving increasing attention. Many studies have indicated that cryopreservation of oocytes and embryos is associated with short-term effects on changes in oocyte epigenetic patterns and long-term adverse health consequences for offspring in both animal models and humans [94,95,96,97]. However, there is little research on these effects in the development of isolated primordial follicles in vitro and in vivo. Therefore, epigenetic stability is expected to be a key focus of future investigations to address safety concerns [98].

### 3.2. Transplantation-Induced Follicle Loss and Its Mechanisms

Female reproductive potential, also known as ovarian reserve, is viewed as a function of the number and quality of remaining follicles [99]. However, follicle loss occurs continuously at each stage, from ovarian tissue removal to transplantation [90]. During the multi-step procedure, mechanical damage, freezing-induced injury during cryopreservation, depletion in bioprosthetic ovary construction, and transplantation-induced follicle loss can all contribute to the overall decline of follicle stock, although the specific contribution of each step can be differentiated. One study proved that there is no significant difference in general follicle growth between frozen–thawed and fresh tissue after long-term xenotransplantation, although the theca layers in antral follicles from frozen–thawed grafts were found to be thinner [100]. These results appeared consistent with another research based on tissue that had been frozen for five years, which also implies that cryopreservation does not strongly contribute to follicle damage [101]. In contrast, the most significant follicle loss is estimated to occur during the early post-graft stage [90,102]. Therefore, preventing transplantation-induced follicle loss becomes a key question to be addressed.

Two major mechanisms contribute to post-transplantation follicle loss: ischemia and activation [90]. Given that ovarian tissue slices are grafted without blood vessels, follicles are exposed to a period of ischemia that persists until complete revascularization. Based on the results of transplantation in chick chorioallantois membrane and severe combined immunodeficient mice, neovascularization of human ovarian tissue appropriately begins on day 3, with full oxygenation and nutrient supply provided by day 10 post-transplantation [103,104,105]. Ovarian cells respond to hypoxia and adapt their oxygen consumption by upregulating the hypoxia inducible factor 1 signaling pathway, which remains active until normal oxygenation is restored [106,107]. The cellular response to hypoxic stress can lead to two potential outcomes: follicular apoptosis and enhanced angiogenesis through the recruitment of proangiogenic growth factors like basic fibroblast growth factor (bFGF) and endothelial progenitor cells [108]. Once oxygen becomes available through vascularization from both the host and the graft, ischemic–reperfusion injury can cause further damage to ovarian cells through complex mechanisms, mainly due to the production of reactive oxygen species and the increased level of local inflammatory mediators [89,109].

**Figure 2:**
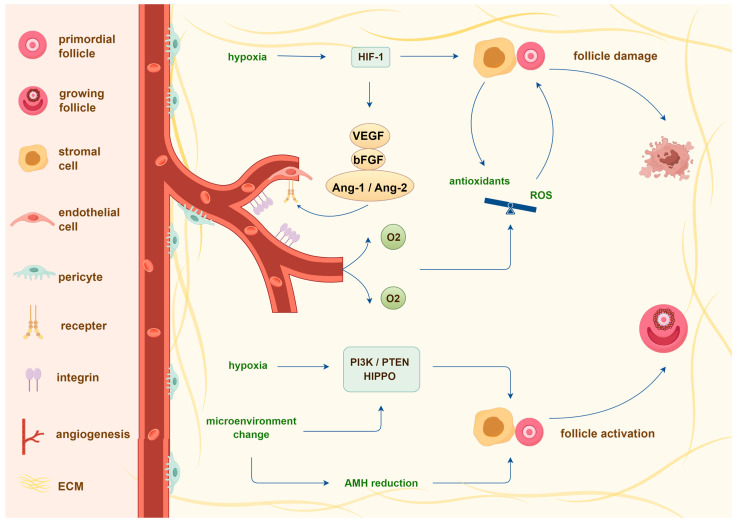
Potential molecular mechanisms underlying transplantation-induced primordial follicle loss. Two mechanisms may be responsible for this effect: ischemia and activation. Hypoxia led by ischemia directly induces cell death and triggers the secretion of angiogenic growth factors such as VEGF, bFGF, and Ang-1/2 via HIF1 signaling activation. As angiogenesis progresses and oxygen becomes available, an imbalance between antioxidants and reactive oxygen species (ROS) generation exacerbates ischemic injury. Additionally, significant activation of primordial follicles is driven by hypoxia, mechanical alterations in the extracellular environment, and the absence of AMH, primarily through the PI3K and HIPPO pathways. Together, these mechanisms contribute to the depletion of the primordial follicle reserve. AMH, anti-Müllerian hormone; Ang-1/2, Angiopoietin-1/2; bFGF, basic fibroblast growth factor; ECM, extracellular matrix; HIF-1, hypoxia inducible factor 1; ROS, Reactive oxygen species; VEGF, vascular endothelial growth factor.

Beyond ischemia, primordial follicle loss is accompanied by a significant increase in larger follicles and granulosa cell proliferation, suggesting that premature recruitment and burnout of primordial follicles occur shortly after transplantation [102,110,111]. Hypoxia and mechanical changes to the extracellular environment are thought to induce abnormal follicle growth through the activation of the HIPPO and PI3K/PTEN signaling pathways [90,102,112,113]. Additionally, this phenomenon might be attributed to the lack of inhibitory factors such as anti-Müllerian hormone (AMH), which are mainly produced by a growing follicle population to maintain the suppression of primordial follicles [114,115]. Compared to primordial follicles, developed follicles are more sensitive to hypoxia and mechanical injury during transplantation. Therefore, the loss of growing follicles might reduce AMH suppression, ultimately resulting in over-recruitment of primordial follicles in grafts [115,116]. Similarly, a previous study showed that the presence of the medulla in addition to the ovarian cortex promotes follicle survival in mouse xenografts, possibly because more developing follicles were preserved [117,118]. Figure 2 summarizes the mechanisms described in the paragraphs above.

## 4. Bioengineering of Bioprosthetic Ovaries to Prevent Transplantation-Induced Follicle Loss

Understanding the mechanisms underlying post-transplantation follicle loss is crucial for identifying pathways that can guide the development of improved therapeutic strategies. To date, numerous growth factors, hormones, and stem cells have been developed and tested to address this problem, mainly via two research directions: reducing ischemic injury and suppressing the over-recruitment of primordial follicles [90]. Additionally, to enhance the localization and sustain concentrations of these bioactive components, bioprosthetic ovary matrix can be bioengineered as biomaterial scaffold-based delivery systems.

### 4.1. Agents Reducing Transplantation-Induced Follicle Loss

#### 4.1.1. Growth Factors

The administration of proangiogenic factors such as VEGF and bFGF is considered promising, with numerous studies demonstrating their effect to improve follicle outcomes in animal models [119,120,121,122]. However, evidence supporting their clinical efficacy in maintaining the human follicle pool remains limited. One study reported that tissue treatment with bFGF enhanced the quality of human ovarian grafts by promoting blood vessel formation and cell proliferation, whereas the addition of VEGF provided no additional benefits [123]. Subsequent research by Tanaka et al. also suggested that sustained release of bFGF induced neovascularization and ultimately increased follicle survival in frozen–thawed human ovarian tissue grafts [124]. In addition to angiogenic agents, several studies have explored strategies to mitigate ischemic damage using cell protectants. N-acetylcysteine (NAC), a widely used antioxidant, has demonstrated potential as a therapeutic agent by preventing follicle loss in mouse ovarian grafts [125]. Regarding human ovarian tissue, Olesen et al. demonstrated the role of NAC as an antioxidant and anti-inflammatory factor in protecting human ovarian reserve from ischemia–reperfusion injury [126]. Other antioxidants, such as erythropoietin and melatonin, have also exhibited protective effects against follicular apoptosis in non-human ovarian tissue. However, evidence of their efficacy in human xenografts remains inconclusive [89].

#### 4.1.2. Hormones

Since abnormal recruitment of primordial follicles is identified as an underlying cause of follicle loss, studies on inhibiting the activation of grafted follicles have been conducted worldwide. As one of the most important suppressors, research on the use of AMH shows controversial results. Experiments on mouse ovarian grafts have provided evidence that AMH supplementation during ovary vitrification and warming can decrease apoptosis, with the effects being influenced by the route of administration [127]. In line with the results from animal experiments, several studies suggest that AMH exhibits complex mechanisms in human ovarian grafts. A study by Man et al. indicated that follicle survival improved when co-transplanted with engineered exogenous endothelial cells, which constitutively expressed AMH and were integrated into a fibrin scaffold [128]. These specialized endothelial cells were found to accelerate vessel perfusion and functionally suppress premature follicle mobilization. Further investigation into the regulatory mechanisms impacting long-term xenografts showed that exogenous AMH also acts at more advanced stages of follicle maturation [129]. However, another study showed that although the administration of recombinant AMH reduced apoptosis and cellular activation during the post-transplant ischemic period, it did not prevent initial follicular depletion [130]. This discrepancy may be attributed to differences in administration approaches and animal models. Alternative molecules may also play a role in inhibiting primordial follicle activation, but few studies have examined their impact on post-graft follicle survival to date.

#### 4.1.3. Stem Cells

Mesenchymal stem cells have been the most extensively studied method with favorable clinical outcomes to date. Research by Xia et al. showed that bone marrow-derived stem cells support ovarian tissue survival during transplantation by increasing blood perfusion and reducing apoptosis [131]. Similarly, another group observed comparable effects with adipose-derived stem cells (ASCs) embedded inside a fibrin matrix on promoting human vessel differentiation, which is probably attributed to increased cell differentiation and VEGF secretion during post-graft stages [132,133]. A subsequent study further showed the long-term benefit of this method in yielding a larger primordial follicle pool after ovarian tissue transplantation [134]. Collectively, these studies indicate that MSCs can regulate follicle reserve through multiple mechanisms during the post-graft stage, including anti-apoptotic effects, enhanced vascularization, and the maintenance of primordial follicles [135]. However, animal studies have also revealed potential side effects associated with ASCs. For instance, research by Damous et al. demonstrated risks associated with the direct injection of ASCs into cryopreserved ovarian grafts in rats, such as impaired morphology and increased apoptosis [136]. A summary of these findings regarding the effects on transplanted follicles is provided in Table 2.

### 4.2. Bioengineering of Bioprosthetic Ovary Scaffolds for Targeted Agent Delivery

Table 2 highlights various administration routes employed for delivering targeted agents in recent studies. Generally, these methods can be classified into two types: systemic delivery, such as direct injection into the host on specific days, and local delivery, which includes incubating grafts in factor-rich media before transplantation or encapsulating agents within scaffolds for co-transplantation. Among these, biomaterial scaffold-based local delivery usually requires lower doses and allows for sustained release of agents, as demonstrated in applications such as bone tissue regeneration and cancer immunotherapy [137,138]. For instance, in two previously mentioned studies, VEGF was embedded into a modified fibrin-based scaffold to reduce degradation and control its release. Encapsulation of bFGF in a gelatin hydrogel extended its bioactivity up to 14 days post-transplantation [119,124]. The short half-life of bioactive factors and hormones often limits adequate contact with the target in systemic delivery. This may partially explain why encapsulating engineered AMH-expressing cells in fibrin outperformed systemic delivery of recombinant AMH via pump in improving follicle survival [128,130]. Moreover, in stem cell therapy, matrix encapsulation demonstrated greater efficiency in sustaining agent delivery to target sites with reduced doses and fewer adverse effects compared to direct graft injection [132,136]. Taken together, these findings underscore the feasibility and advantages of biomaterial scaffold-based local delivery in mitigating transplantation-induced follicle loss. Additionally, the emergence of new technologies may make the bioengineering of bioprosthetic ovaries more available and efficient.

### 4.3. Future Directions on Bioprosthetic Ovary Construction

Emerging technologies with potential applications in the field of female fertility preservation and restoration include 3D-printed ovaries, ovarian tissue papers, and microfluidic culture models. As we have previously mentioned, the 3D-printed ovary is an innovative experimental technology of the bioprosthetic ovary in which isolated ovarian follicles are embedded in 3D-printed microporous hydrogel scaffolds to support further follicular growth after transplantation in vivo. A groundbreaking study by our coauthor, Teresa K. Woodruff, and her research team demonstrated complete restoration of endocrine and reproductive ovarian function after xenografting bioprosthetic ovaries composed of 3D-printed, follicle-seeded scaffolds into surgically sterilized mice. Following natural mating, these mice successfully conceived and produced healthy offspring [69]. This performance has been confirmed in more subsequent studies as well [65,70]. More recently, tissue papers derived from organ-specific decellularized extracellular matrices have been explored for their potential to support folliculogenesis. In another pivotal study by Woodruff’s group, ovarian tissue papers supported the adhesion, viability, and overall health of mouse ovarian follicles in vitro. Furthermore, these tissue papers sustained the viability and hormonal function of live ovarian cortical tissues containing follicles from nonhuman primates and humans in ex vivo culture. Tissue papers can be further augmented with additional synthetic and natural biomaterials, and integrated with recently developed, advanced 3D-printed biomaterials, providing a versatile platform for future multi-biomaterial construct manufacturing [139]. Additionally, microfluidic technology is regarded as a promising new strategy to stimulate the dynamic flow conditions of natural organs. Another study by Woodruff’s team demonstrated that microfluidic culture models can replicate the human reproductive tract and simulate a 28-day menstrual cycle, offering a novel platform for studying cell–microenvironment interactions in ovarian tissue [140]. Overall, the promise of these future directions may facilitate the clinical translation of bioprosthetic ovary technologies, offering new avenues for restoring female fertility in both cancer and non-cancer patients—particularly when conventional fertility preservation and restoration methods are infeasible or contraindicated [141].

Conversely, the application of these emerging technologies may also raise regulatory and ethical concerns. In addition to bioprosthetic ovaries, biomaterial-based engineered tissues and organs have become essential elements in various biomedical fields. In general, the regulation of these artificial biomaterials should involve rigorous preclinical testing, clinical trials, and post-marketing surveillance to ensure their safety and efficacy [142]. However, in practice, these regulatory processes are highly complex, and major agencies in different regions—such as the U.S. Food and Drug Administration (FDA) and the European Medicines Agency (EMA)—adopt varying standards and assessment criteria for evaluating the safety and effectiveness of biomaterials [142]. Therefore, the establishment of a regulatory standard that is both broadly applicable and practically implementable should be considered a key issue for future discussion. Moreover, all stages involved in the development of biomaterials—including subject recruitment, risk–benefit assessment, and informed consent—should be conducted in accordance with established ethical principles [143]. The commercialization of biomaterial-based reagents may also raise ethical concerns related to donor consent, privacy, and the sustainability of production [143]. In conclusion, the incorporation of regulatory oversight and ethical standards into the development of bioprosthetic organs and tissues is essential for advancing the field responsibly while ensuring fairness and equity in human health.

## 5. Conclusions

Bioprosthetic ovaries provide an alternative way to restore fertility in cancer patients who are unsuitable candidates for direct transplantation of cryopreserved ovarian tissue. Moreover, bioprosthetic ovary scaffolds are studied as vehicles for the delivery of bioactive factors, hormones, and stem cells to reduce transplantation-induced follicle loss. Meanwhile, concerns regarding the safety and efficacy of bioprosthetic ovary construction deserve further attention, as they represent central questions in understanding how follicle development and revascularization are influenced by the extracellular matrix. Further research on the construction and bioengineering of bioprosthetic ovaries could provide valuable insights into the processes of folliculogenesis and cell–matrix interactions. This technology also has the potential to serve as a platform for studying the effects of various drugs on human fertility, including the development of anti-apoptotic agents and the toxicity testing of chemotherapeutics. Furthermore, advancements in bioprosthetic ovary construction may benefit from emerging innovations in tissue engineering, such as 3D printing and microfluidic technologies. In conclusion, the bioprosthetic ovary technique has the potential to advance interdisciplinary research at the intersection of biomedicine, polymer chemistry, and material science.

## Figures and Tables

**Table 1:** Matrix used to graft isolated mouse or human follicles and their main outcomes.

Matrix	Graft Species	Graft Duration	Outcomes	Studies
plasma clots	mouse	up to 15 weeks *	offspring	[41]
plasma clots	mouse	up to 12 weeks	offspring	[42]
plasma clots	human	7 days	FS (20%); FD	[43]
plasma clots	human	5 months	FS (29%); FD	[44]
alginate	mouse	1 week	FS (35%); FD	[63]
alginate	mouse	1 week	FS (20%); FD	[64]
fibrin	mouse	7 days	FS (32%); FD	[47]
fibrin	mouse	21 days	FS (17%); FD; HP	[51]
fibrin with VEGF	mouse	up to 6 months	HP; offspring	[53]
fibrin	mouse	7 days	FS (28%); FD	[52]
fibrin	human	7 days	FS (23%)	[55]
fibrin	human	up to 7 days	FS (35%); FD	[23]
fibrin with platelet lysate	mouse	2 weeks	FS (67%); FD	[54]
dECM from bovine ovary	mouse	up to 4 weeks	HP	[78]
dECM from human ovary	mouse	3 weeks	FS (21%); FD	[79]
dECM from human ovary	human	3 weeks	FS (25%)	[79]
gelatin	mouse	up to 10 weeks *	HP; offspring	[69]
PEG-VS	mouse	60 days	FD; HP	[83]

* Calculated based on the figure shown in the studies. dECM, decellularized extracellular matrix; FS, follicle survival, numbers in parentheses indicate the proportion of follicle survival; FD, follicle development; HP, hormone produced; PEG-VS, poly(ethylene glycol) vinyl sulfone.

**Table 2:** Agents have been validated to improve transplanted follicle outcomes.

Substance	Graft Species	Administration Routes	Graft Duration	Outcomes	Studies
VEGF	mouse	encapsulation in fibrin	up to 20 days *	increased FS; offsprings	[119]
bFGF	mouse	encapsulated in fibrin	1 week	increased RV, FS; decreased AP	[120]
VEGF	sheep	embedded in collagen	up to 3 weeks	Increased FS	[121]
bFGF& VEGF	mouse	encapsulated in fibrin	up to 21 days	increased RV, FS; decreased AP	[122]
bFGF	human	tissue culture before grafting	7 days	increased RV, FS; decreased AP	[123]
bFGF	human	encapsulation in gelatin	6 weeks	increased RV, FS	[124]
NAC	mouse	host treatment	28 days	decreased AP; increased FS	[125]
NAC	human	host treatment	4 weeks	decreased FA; increased RV, FS	[126]
AMH	mouse	supplemented during warming	28 days	decreased AP	[127]
AMH	human	co-transplanted with AMH-expressing cells	2 weeks	decreased FA; increased RV, FS	[128]
AMH	human	host treatment	14 days	decreased AP, FA	[130]
AMH	human	co-transplanted with AMH-expressing cells	up to 14 weeks	decreased AP, FA	[129]
ASCs	mouse	inject into graft	30 days	increased AP	[136]
BSCs	human	embedded in Matrigel	21 days	increased RV, FS; decreased AP	[131]
ASCs	human	embedded in fibrin	7 days	increased RV, FS	[132]
ASCs	human	embedded in fibrin	6 months	increased FS	[134]
ASCs	human	embedded in fibrin	10 days	decreased AP, FA	[135]

* Calculated based on the figure shown in the studies. AMH, Anti-Mullerian hormone; AP, apoptosis; ASCs, adipose-derived stem cells; bFGF, basic fibroblast growth factor; BSCs, bone-marrow-derived stem cells; FA, follicle activation; FS, follicle survival; NAC, N-acetyl cysteine; RV, revascularization; VEGF, vascular endothelial growth factor.
